# Mild cognitive impairment understanding: an empirical study by data-driven approach

**DOI:** 10.1186/s12859-019-3057-1

**Published:** 2019-12-24

**Authors:** Liyuan Liu, Bingchen Yu, Meng Han, Shanshan Yuan, Na Wang

**Affiliations:** 1 Data-driven Intelligence Research Laboratory, Kennesaw State University, 1100 South Marietta Pkwy, Marietta, GA USA; 2 Georgia State University, 33 Gilmer Street SE, Atlanta, 30302 GA USA; 3 Hubei University, 11 Xueyuan Road, Wuhan, 430062 Hubei China; 4 Key Laboratory of Systems Biomedicine (Ministry of Education) and Collaborative Innovation Center of Systems Biomedicine, Shanghai Center for Systems Biomedicine, Shanghai Jiao Tong University, 800 Dongchuan Road, Shanghai, 200240 China

**Keywords:** Mild cognitive deline impairment (MCI), Data-driven approach, Machine learning

## Abstract

**Background:**

Cognitive decline has emerged as a significant threat to both public health and personal welfare, and mild cognitive decline/impairment (MCI) can further develop into Dementia/Alzheimer’s disease. While treatment of Dementia/Alzheimer’s disease can be expensive and ineffective sometimes, the prevention of MCI by identifying modifiable risk factors is a complementary and effective strategy.

**Results:**

In this study, based on the data collected by Centers for Disease Control and Prevention (CDC) through the nationwide telephone survey, we apply a data-driven approach to re-exam the previously founded risk factors and discover new risk factors. We found that depression, physical health, cigarette usage, education level, and sleep time play an important role in cognitive decline, which is consistent with the previous discovery. Besides that, the first time, we point out that other factors such as arthritis, pulmonary disease, stroke, asthma, marital status also contribute to MCI risk, which is less exploited previously. We also incorporate some machine learning and deep learning algorithms to weigh the importance of various factors contributed to MCI and predicted cognitive declined.

**Conclusion:**

By incorporating the data-driven approach, we can determine that risk factors significantly correlated with diseases. These correlations could also be expanded to another medical diagnosis besides MCI.

## Background

**Fig. 1:**
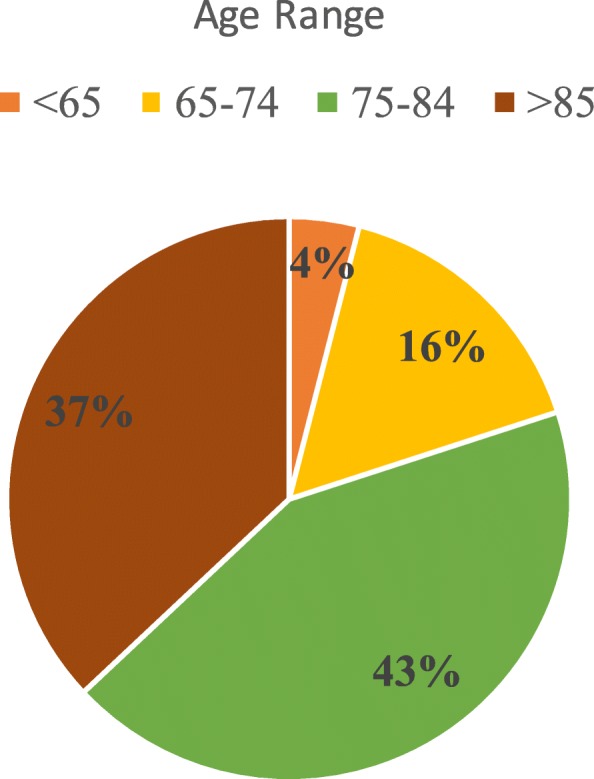
Percentage of Dementia/Alzheimer by Age

Cognitive decline has emerged as a significant threat to both public health and personal welfare [1–5]. The number of dementia cases worldwide has been estimated to be more than tripled by 2050 compared with 2010 [6]. An estimated 5.5 million Americans of all ages are living with Dementia/Alzheimer disease in 2017. This number carries an estimated 5.3 million people age 65 and older, and approximately 200,000 individuals under age 65 who have younger-onset Dementia/Alzheimer disease, though there is more significant uncertainty about the younger-onset estimate [7]. Figure 1 is the Alzheimer’s disease distribution by age, which clearly shows that this disease poses a great threat to the elder. With the gradually increased lifespan, this disease will continue to be a major concern to public health.

While the development of effective anti-dementia drugs and therapeutic procedures are in high demand, this process can be time, resource consuming and many efforts have ended up with ineffective [2, 6]. What is at least on par important with development anti-dementia drugs is the identification of modifiable risk factors which contribute to cognitive decline/dementia [5]. These are especially true with MCI [8]. The symptom of MCI is a decrease in memory, attention, and cognitive function that beyond what would be considered reasonable based on the individual’s age and level of education. Although MCI would not significantly affect the daily living and social activities, it is a sign of an early stage of Alzheimer type dementia, and many patients transfer from MCI to Dementia/Alzheimer’s disease within few years. Evidence showed that the exclusion of modifiable risk factors could reduce cognitive decline risk [8, 9]. For example, physical excise showed a protective effect in MCI while smoking increases the risk of MCI. Thus, the identification of MCI modifiable risk factors can play a significant role in both understanding the mechanism of cognitive decline and prevention of cognitive decline [6]. The identification of MCI modifiable risk factors can prevent of MCI or at least decrease the MCI risk, which will further attenuate the threat of dementia and Alzheimer’s disease and provide a complementary strategy to the development of anti-dementia drugs and therapeutic procedures.

In the process of discovery of modifiable risk factors for MCI, well-controlled lab experiment and clinical conservation played an important role and laid a foundation in this area [3]. However, this approach is time and resource consuming and has a limited risk factor studied at one time. On the other hand, modifiable risk factors contribute to MCI could potentially be numerous, considering the complicity of cognitive and its related pathways [3]. Thus, a new strategy and angel to look into this issue is in great need. Previous survey and studied has identified several modifiable risk factors such as hypertension, diabetes mellitus, hyperlipidemia, chronic renal failure, reduced physical activity, alcohol consumption, and smoking. Despite the break-through mentioned above, there is still a great need to exam the reliability of previously found risk factors and explore more risk factors. For example, some conclusions are still controversial, such as the linkage between cognitive decline and diabetes mellitus. Another potential issue of many previous results is that they are based on limited samples.

Data science have started with statistics, along with computer science, including the concepts/practices such as artificial intelligence, data mining, and machine learning, *etc*. Data science become a more and more attractive discipline. There are many researchers use advanced statistical methods to discover real-world problems in the bioinformatics research area. Cai et al. did a lot of model-based researches related to bioinformatics [10–12]. For example, they proposed a temporal model to reduce the inherent temporal bias of hemagglutination inhibition tables caused by herd immunity [13]. Bayesian, as another model-based model, is one of the most important methods widely used in bioinformatics research [14]. The data-driven approach as a potent tool to solve many emerging scientific challenges, also roots of data and drives the answer to the natural question of real life. Different from the traditional research approach, data-driven way allows the fact of discovery even before the hypothesis, and the last step is not verification but reasoning the behind knowledge.

Over the past decades years, data-driven approach has increased on a large scale in various fields. As more and more data has become available, first by way of recorded behaviors and trends, biology, chemistry, and then many other traditional domains have been collecting and storing it in ever more significant amounts. With the growth of the Internet, the exponential growth of data volumes become publicly available to researchers; there has been a flood of new information, and big data. There are many researchers used data-driven approach in advanced technology domains, including enterprise management [15], Internet of Things [16], online social networks [17, 18], social networks influence [19], traffic monitoring [20], media applications, collective intelligence and data pravicy [21, 22]. Machine learning and deep learning are popular research topics recently. They can figure out how to perform essential tasks by generalizing from voluminous examples. Bioinformatics research is characterized by full or incremental datasets and sophisticated data analytics methods [23]. Therefore, machine learning and deep learning are universally applied in bioinformatics research. Many researchers used advanced artificial intelligence to predict or forecast the real-world problems, the more related studies can be found in [24–26].

In this study, we intended to use a data-driven approach along with the machine learning techniques to analyze a large amount of collected health data to:

- Exam the previously discovered modifiable risk factors;
- Find new modifiable risk factors contribute to MCI;
- Build an index reflecting the weight and contribution of various factors.

The rest of the paper is organized as follows. The background and the motivation, along with the related works, are introduced in Introduction. “Methods” section illustrates the preparation of our research data, the data analysis, and visualization. The evaluation results and open the discussion of our contribution are demonstrated in the “Results and discussion” section. We discuss the conclusion in the “Conclusion” section.

## Methods

### Data source

The data used was collected from Centers for Disease Control and Prevention (CDC) based on Behavioral Risk Factor Surveillance System (BRFSS). BRFSS was initiated by CDC in 1984 to conduct the monthly survey over landline telephones and cellular telephones. This survey collected data among adult U.S. residents regarding their risk behaviors, daily lifestyle, physical/mental health condition, and care or insurance status. It is a nationwide survey system covering all the states, the District of Columbia, and participating U.S. territories with more than 500,000 interviewees attending yearly. This survey includes standard core questions, which is asked by all states and optional modules, which is selectively wondered by some states.

We are using the 2016 survey data (https://www.cdc.gov/brfss/annual_data/annual_2016.html). Questionnaire related to cognitive decline belongs to “Optional Modules” and was used by more than 20 states nationwide including Alaska, Delaware, Idaho, Indiana, Kentucky, Massachusetts, Missouri, Montana, New Hampshire, et al. The data used in this study was based on the primary data collected by those states. For states participate in the “cognitive decline modules,” interviewees who are older than 45 were asked several questions related to their current cognitive status. Therefore, data from those states include each the cognitive status participates (who are more than 45 years old) together with physical/mental health condition such as diabetes, cancer, stroke and daily lifestyle such as cigarette usage, alcohol consumption, exercise amount, which will allow us to analyze and verify any correlation between those factors and MCI.

### Data organization and cleaning

The factors we choose to study that may contribute to MCI was extracted from the primary data using Python and were characterized as five categories: Health/disease condition (general physical/mental health condition, skin cancer, diabetes, arthritis, stroke, heart condition, asthma, kidney disease, pulmonary disease), daily lifestyle (alcohol consumption, cigarette usage, exercise amount, sleep time), emotion (depression, children amount), health care accessibility (whether have health insurance, frequency of visiting doctor), education level and some other factors (whether a veteran, rent or own a house, amount of removed tooth). Cognitive decline was rated from 0–0.5, where 0 stands for no sign for MCI based on the question “have you experienced confusion or memory loss that is happening more often or is getting worse”. 0.1 to 0.5 represent the extent of cognitive decline based on the question “does confusion or memory loss interfere with work or social activities?” with 0.5 represent the most severe decline. It is worth clarifying that BRFSS collected the data through the telephone-based questionnaire, which means the interviewees are capable of picking up the phone and answer the questions with no doubt. With this ability, they would fall into the MCI category if they showed any sign of cognitive decline based on their answer. Data with the interviewees’ answer is “Don’t know/Not sure” and “Refused” was removed. In this study, 60816 valid interviewee data was obtained after clean up the primary data.

### Data analysis and visualization

Before the modeling phase, to prevent the over-fitting problem and improve model performance, 5-fold cross-validation is used to split data into training and validation datasets. In 5-fold cross-validation, the original dataset is randomly partitioned into five equal-sized sub-datasets. Of the five sub-datasets, a single sub-dataset is retained as the validation data for testing the model, and the remaining four sub-datasets are used as training data. The cross-validation process is then repeated five times, with each of the five sub-datasets used exactly once as the validation data. The five results from the folds can then be averaged to produce a single estimation.

**Fig. 2:**
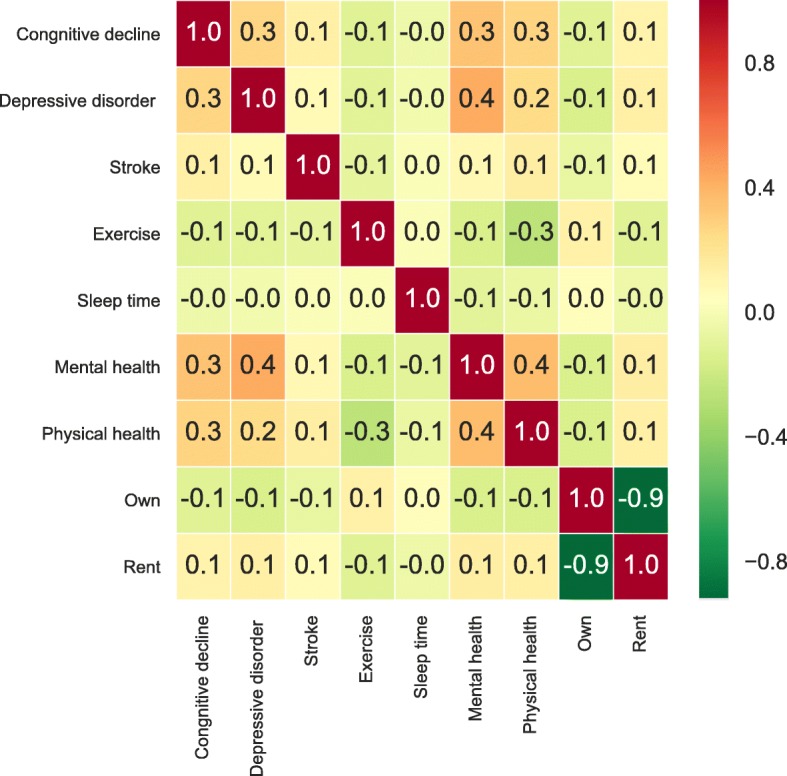
Correlation of Various Factors

The correlation matrix is used to investigate the dependence between multiple variables at the same time. There are several different methods for correlation analysis: Pearson parametric correlation test, Spearman, and Kendall rank-based correlation analysis. Pearson correlation coefficient is the method employed to measure the linear dependence between two variables. It has values between positive 1 and negative 1, where 1 is the total positive linear correlation, and 0 is no linear correlation, and negative 1 is the total negative linear correlation. Figure 2 is the Pearson parametric correlation matrix of selected variables.

### Oversampling and undersampling

**Fig. 3:**
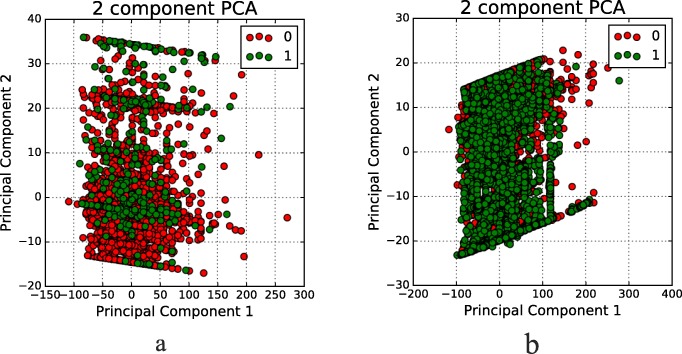
Before and After Oversampling and Undersampling **a** Classes Before Oversampling and Undersampling; **b** Classes After Oversampling and Undersampling

The class-imbalanced problem usually is existing in bioinformatics research. This problem can get worse when the class of interest is regularly the minority class. Synthetic Minority Over-sampling Technique(SMOTE), as an efficiency technology is aimed to solve the class-imbalanced problem. It is developed by Chawla et al. that combined the techniques of over-sampling the minority (abnormal) class with the under-sampling the majority (normal) class. Regarding [27], SMOTE could achieve better classifier performance in ROC space. In this study, we employ SVM-SMOTE to balanced the dataset. SVM-SMOTE uses the SMOTE algorithm to generate more false-positive samples and then builds an SVM on the oversampling dataset [28]. There is an essential parameter in SVM-SMOTE denotes *R*_*o*_. If there are *N*_*positive*_ positive samples, we should add *R*_*o*_∗*N*_*positive*_ pseudo positive samples into the initial training dataset; then the grid search will determine the optimal value of *R*_*o*_. The reason why we use random under-sampling as our primary technique could be found in Dittman [29]. It shows random under-sampling presented the most common top-performing data sampling technique and more computationally cheap. Figure 3 shows a sample visualization of training data that before or after oversampling and undersampling. Oversampling and undersampling have balanced the classes in the training dataset.

### Gradient boosting

In general, gradient boosting tree concerns to a family member of decision tree learning which drawing observations to conclusions about the target value in a tree structure. As an ensemble learning algorithm, each decision tree is trained based on the performance of the previous trees. Regarding the variable target characteristics, it can be used for regression or classification purpose. In the gradient boosting learning problems, there is a learning set *L*={(*X*_1_,*Y*_1_),…,(*X*_*n*_,*Y*_*n*_)} of known values of X and corresponding label values of Y, the goal is to find an approximation F(X) to a function F(X) that minimizes the expected values of some specified loss function L(Y,F(X)) [30].

1 $$ \tilde{F}=\underset{F}{\operatorname{argmin}}\mathbb{E}_{X,Y}[L(Y,F(X))]  $$

More background of gradient boosting trees can be reached in Chen’s research [31]. In this study, the gradient boosting tree is typically used with decision trees, especially for CART trees. Gini index is used to determine the candidate variables for splitting each node in each decision tree model.

### Random forests

**Fig. 4:**
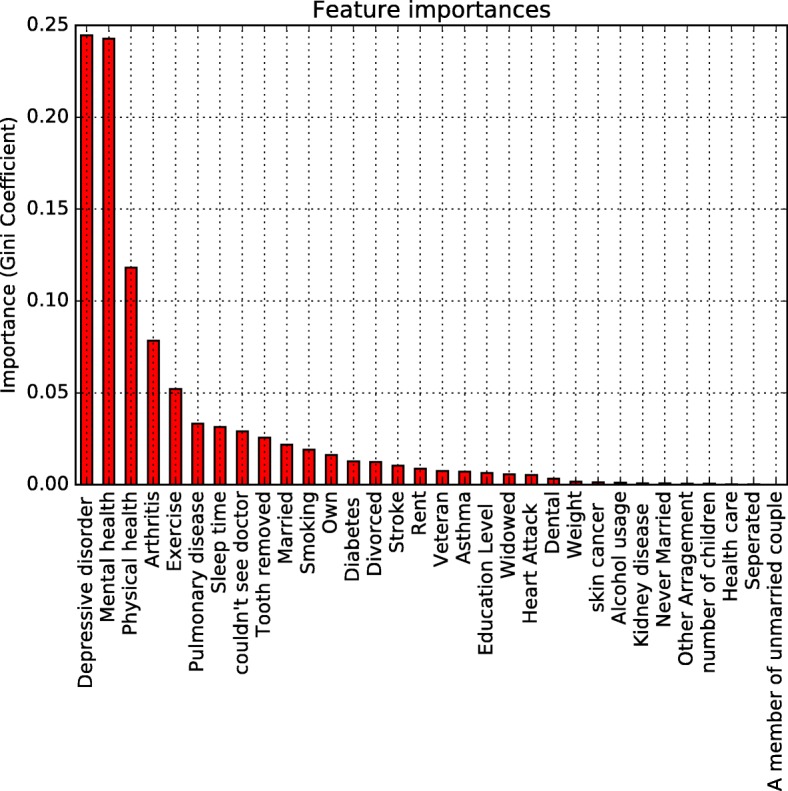
Weighed Feature Importance Contributing MCI

It is a combination of tree predictors such that each tree depends on the values of a random vector sampled independently and with the same distribution for all trees in the forests [32]. In the classification problem, a learning set denotes to *L*={(*X*_1_,*Y*_1_),…,(*X*_*n*_,*Y*_*n*_)} and n observations of a random vector (X, Y). Vector *X*=(*X*^1^,…,*X*^*m*^) contains dependent variables that $X\in \mathbb {R}^{m}$, $Y\in \mathcal {Y}$, $\mathcal {Y}$ is a target value. For the classification problems, a classifier t is a mapping t: $\mathbb {R}^{m}\rightarrow \mathcal {Y}$ while for regression problems, *Y*=*s*(*X*)+*ε* with E[ *ε*|*X*]=0 and s is called regression function [31]. Random forests are the model provided estimators of the Bayes classifier and regression function, for the classifier purpose, they support minimizing the classification error *P*(*Y*≠*t*(*X*)). CART model and bagging are two popular tree-based methods to be used in random forests. More details of random forests background can be found in Hastie’s research [33]. A random forest model is created from the weighted or unweighted average predicted values of all the decision trees. Same as gradient boosting tree, we employ Gini index to split the nodes of each decision tree. We also generated the importance of the factors could be ranked based on the Gini reduction as Fig. 4 showed.

### Logistic regression

Logistic regression presents a method for modeling a binary response variable; the labels valued 0 and 1. The most significant benefit to use logistic regression is when we have a classification problem, we can manage more than two explanatory variables simultaneously. Logistic regression will model the probability of an outcome based on individual characters; the relationship between the input set *X*_1_,*X*_2_,…,*X*_*n*_) and the predicted probability P of the classes can be defined as:

2 $$ log\left(\frac{P}{1-P}\right)=\beta_{0}+\beta_{1}X_{1}+\dots+\beta_{n}X_{n}  $$

We use L2 regularization with primal formulation to prevent the multicollinearity problem in this study.

### Neural network

The neural network is a general method of regression and classification. We train the model using backpropagation with four layers. A linear combination activation function ReLU worked in second and third layers, and binary classification activation function sigmoid used in the last layer. Dropout is an efficient technique for preventing over-fitting in deep learning. Unlike standard weight regularizers, such as based on the L1 or L2 norms, that push the weights toward some expected prior distribution [34]. So we add a dropout dense after each layer. According to previous research [35], authors found that the activation function ReLU could significantly speeding up network training over traditional sigmoidal activation functions, such as tanh; we use ReLU as the in the second and third layers. The sigmoid function is operated in the prediction layer. The Relu (Eq. 3) and sigmiod (Eq. 4) functions are shown as:

3 $$ f(X)=max(X,0)  $$

4 $$ sigmoid(X)=\frac{e^{X}}{e^{X}+1}  $$

### Evaluation metrics

Computing just the accuracy score for a classification model gives a half-done view of the model’s performance. There are much other evaluation metrics, such as the confusion matrix, ROC curve, precision, and recall. In this study, we use accuracy, ROC AUC, recall, and precision as our evaluation metrics. Recall, also called sensitivity, can be achieved by the Eq. 5.

5 $$ Recall=\frac{True Positive}{True Positive+False Nagetive}  $$

Precision is the positive predictive value which can be get by the calculation 6:

6 $$ Precision=\frac{True Positive}{True Positive+False Positive}  $$

The performances of the four models are evaluated by the defined metrics. And the result will be discussed in the “Results and discussion” section.

## Results and discussion

**Fig. 5:**
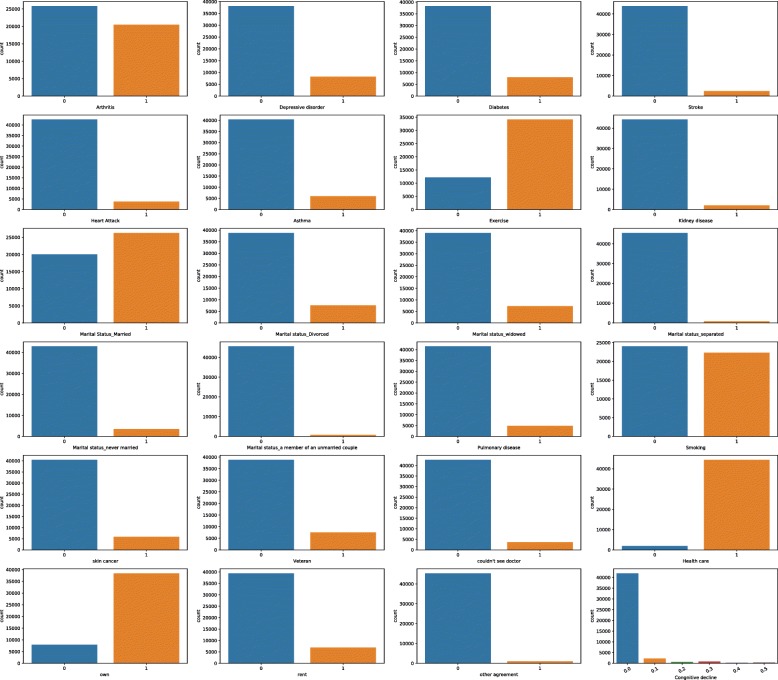
Distribution of Various Factors

Before we analyze the potential correlation between the various factor and cognitive decline, we examine the distribution of each factor first. Form the distribution results, as shown in Fig. 5, most of the interviewees do not have cognitive decline/impairment, which is consistent with their daily life experience and observation. For each rare disease condition such as general physical/mental health condition, skin cancer, diabetes, arthritis, stroke, heart condition, asthma, kidney disease, pulmonary disease most of the interviewees remain health, which also consistent with the real-world situation.

### Cognitive decline analysis

**Fig. 6:**
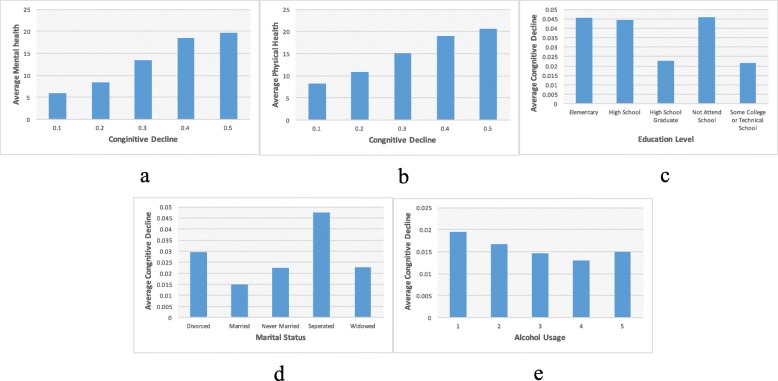
Correlation Between Cognitive Decline and Single Modifiable Risk Factors **a** Mental Health; **b** Physical Health; **c** Education Level; **d** Marital Status; **e** Alcohol Usage

General mental health condition was based on the questionnaire “Now thinking about your mental health, which includes stress, depression, and problems with emotions, for how many days during the past 30 days was your mental health not good?” Fig. 6 shows the correlations between cognitive decline with some potential factors. The more amount of days with poor mental health issues, the higher cognitive decline scores as Fig. 6a showed.

For people without MCI, the average days have the poor mental condition is 2.42, while people have a cognitive decline score of 0.5, the average days have poor mental conditions is 19.46. We observed a strong correlation between general mental health and cognitive decline with the Gini coefficient between mental health and cognitive decline is 0.23. The correlation score between those two factors is 0.3. However, we would not conclude that poor mental health condition is a critical risk factor contribute to cognitive decline because on the contrary, it is very likely the declined/impaired cognitive ability lead to the negative emotion such as stress, depression.

Instead, we use this strong correlation as an indication of the validity of our data process and analysis because the correlation between MCI and mental health is strongly supported both by academic research and daily observation. Our analysis also showed the correlation between MCI and mental health, which prove the accuracy of our data analysis method.

General physical health condition was based on the questionnaire “Now thinking about your physical health, which includes physical illness and injury, for how many days during the past 30 days was your physical health not good?” A strong correlation between the extent of cognitive decline and the number of days that have poor physical health was observed.

For people did not show sign of any cognitive decline (cognitive decline score is 0), the average day of poor physical health is 2.42, while this value increases to 19.46 with people who experience the severe cognitive decline (cognitive decline score is 0.5). We also saw the clear trend between increased amount of days (from 6.10–19.46) that have poor physical health with cognitive decline score (from 0.1 to 0.5) as Fig. 6b showed.

This data and correlation suggested that poor physical health is essential to risk factors that contribute to MCI. The Gini coefficient between physical health and cognitive decline is 0.125 (Fig. 4). This finding is consistent with previous literature that poor physical health contributes to MCI [2, 4].

Depressive disorder condition was based on the questionnaire “Ever told you that you have a depressive disorder, including depression, major depression, dysthymia, or minor depression?” Based on the feather importance analysis, depressive disorder being the most critical risk factor contributing to MCI with a Gini coefficient being 0.24 (Fig. 4). Depression has been long known to be a risk factor that can lead to MCI [4]. In our study, by the analysis of various data at once, we can weigh the importance of different factors. What is new in this finding is that depression is an essential feature among all the features that we studied, it is more important than physical health, health disease, sleep time or cigarette usage, which was traditionally considered significant features.

Education level was based on the questionnaire “What is the highest grade or year of school you completed?” There is a clear correlation between education level and cognitive decline (Fig. 6c). It shows the rule that the higher education level, the smaller cognitive decline score. However, we don’t think a lack of education directly contribute to cognitive decline, it is possible the people with higher education degree tend to live a higher quality of life, such as less suffering from poverty, better access to health care, more opened mind, those factors derived from education level contribute to cognitive decline status. From the feature importance analysis, among all the features analyzed, education level played a moderate role with Gini coefficient being 0.025 (Fig. 4), much less than depression, physical health, cigarette usage, and another disease.

There is an interesting correlation between marital status and cognitive decline. Married people tend to have a lower cognitive decline score (Fig. 6d), while “separated” and “divorced” people tend to have much higher scores, which means a more severe cognitive decline. Form these analyses; we can conclude that a successful marriage can reduce the risk of MCI. Alcohol consumption was based on the questionnaire “During the past 30 days, how many days per week or month did you have at least one drink of any alcoholic beverage such as beer, wine, a malt beverage or liquor?” Interestingly, people without the sign of cognitive decline or have a low score of cognitive decline tend to have more alcohol consumption than those who have a higher score of cognitive decline. Previous studies showed that moderate consumption of alcohol could reduce the risk of MCI [2].

Many existing health conditions such as arthritis, pulmonary disease, stroke, asthma also contribute to MCI risk [8, 9]. There has been a debate about whether diabetes is a risk factor to MCI; some pieces of evidence support this conclusion while others showed no correlation between diabetes and MCI [36]. Based on our analysis, we found that diabetes is a risk factor to MCI but with very moderate influence, much weaker than some other factors such as depression, physical health, arthritis, pulmonary disease, stroke, asthma, and marital status, *etc.*

### Models performance comparison

Table 1 shows the results of accuracy, recall, precision, and ROC AUC of the four machine learning algorithms. Concerning accuracy, all four machine learning models can reach an accuracy higher than 78%. Even though neural networks return the lowest accuracy, it has the highest recall and ROC AUC values which we are more interested becasue they are related to the correct positive prediction.

**Table 1:** Models Evaluation

Models Evaluation				
Models	AUC	Recall	Precision	Accuracy
Gradient Boosting	0.64	0.32	0.41	0.89
Random Forests	0.69	0.49	0.31	0.84
Logistic Regression	0.70	0.56	0.27	0.81
Neural Networks	0.71	0.59	0.30	0.79

### Combined variables comparison

To find the hidden correlation between the risk factors to MCI, we combined different risk factors and compared the models’ performance. The purpose of this experiment is to find how different combination of risk factors can affect the MCI. In general, remove as much as the most significant risk factors will decrease models’ performance more. However, the results show that in some combinations, a more factors combination will have fewer risks to MCI than fewer numbers of a combination. We evaluate the performance as accuracy, recall, precision, and ROC AUC. We choose the five most important risk factors: Depressive disorder(D), Mental health(M), Physical health(P), Arthritis(A), and Exercise(E).

**Fig. 7:**
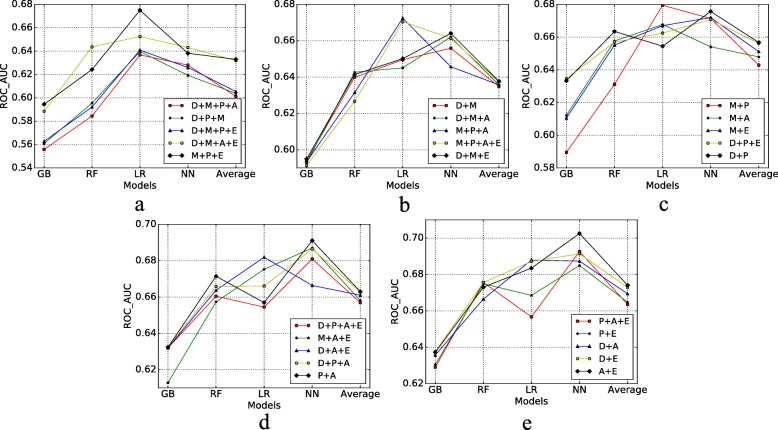
AUC of Different Risk Factor Combinations **a** AUC 1; **b** AUC 2; **c** AUC 3; **d** AUC 4; **e** AUC 5

**Fig. 8:**
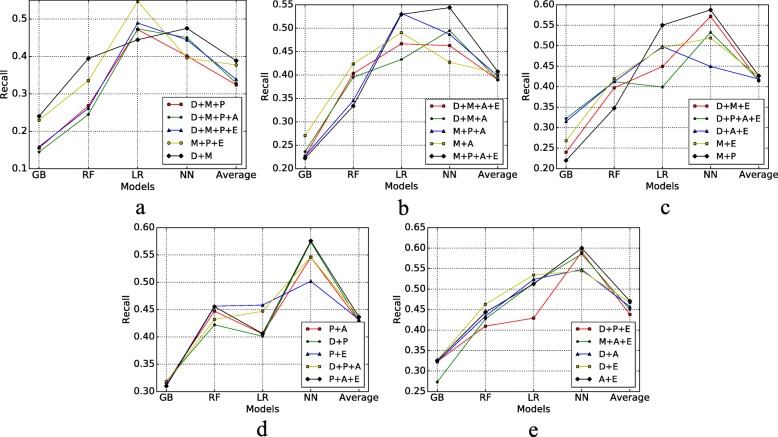
Recall of Different Risk Factor Combinations **a** Recall 1; **b** Recall 2; **c** Recall 3; **d** Recall 4; **e** Recall 5

**Fig. 9:**
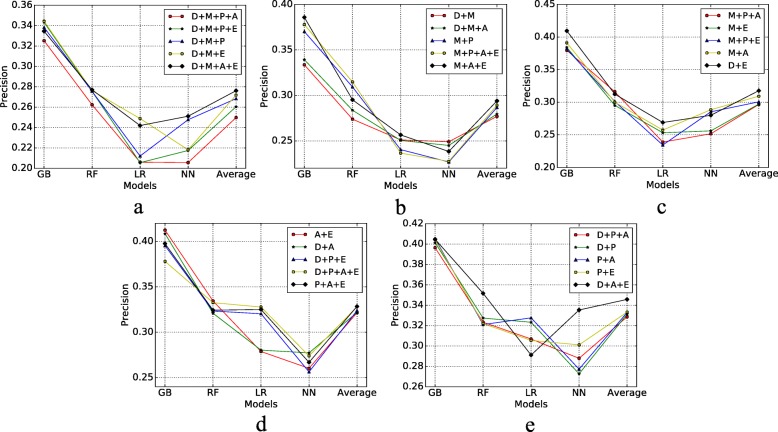
Precision of Different Risk Factor Combinations **a** Precision 1; **b** Precision 2; **c** Precision 3; **d** Precision 4; **e** Precision 5

**Fig. 10:**
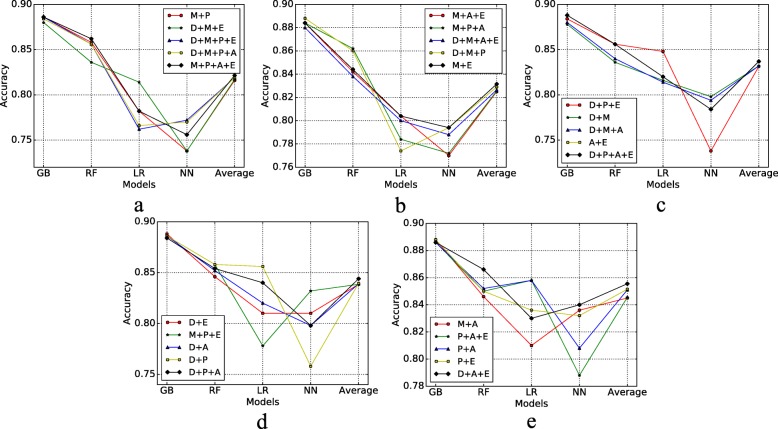
Accuracy of Different Risk Factor Combinations **a** Accuracy 1; **b** Accuracy 2; **c** Accuracy 3; **d** Accuracy 4; **e** Accuracy 5

Figure 7 shows the ROC AUC results of different risk factors combined with four models, also we calculate the average evaluation values of the four models to compare the combination effects. In this figure, there are many insights; for example, we can find that Depressive disorder+Physical health+Arthritis+Exercise has less risk to MCI compare with Depressive diorder+Physical health+Exercise. Figure 8 displays the recall of different risk factor combinations for four different models. Figure 9 shows the precision and Fig. 10 shows the accuracy. From the experiment results, we found that many combinations show the conclusion that in the same situations. More factors combinations will have fewer risks to MCI than fewer numbers of combinations.

### Cluster analysis

Cluster analysis has become the standard tool for bioinformatics researcher. It is employed as a classification tool for unsupervised learning. Some researchers have used it as a means of representing the structure of data via the construction of dendrograms [37]. In bioinformatics, cluster analysis can group multiple observations based on the characteristics of individual’s phenotypes into a series of clusters and help build a taxonomy of groups and subgroups of similar plants. In this study, we employ one of the most popular clustering method *k*-means, which is an incremental approach to clustering, and it is well known for its efficiency. The idea is aiming to minimize the sum of squared distances between all points and the cluster center.

We employed the 33 variables to cluster the observations. Before the clustering, standardization is a standard requirement since the data might misbehave if the particular feature does not more or less look like standard customarily distributed data [38]. Then we determine “K” value using Elbow curve. The Elbow method is a method of interpretation and validation of consistency within-cluster analysis and help researchers finding the appropriate number of clusters. The more background of this method has been described in Tibshirani’s paper [39].

**Fig. 11:**
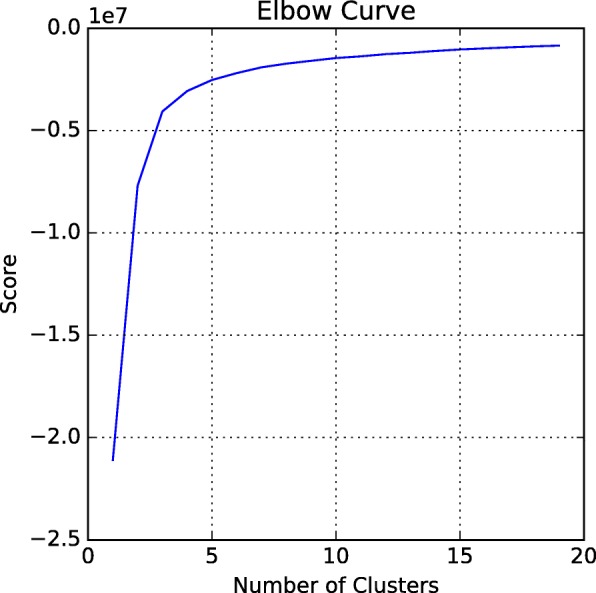
Elbow Method

**Fig. 12:**
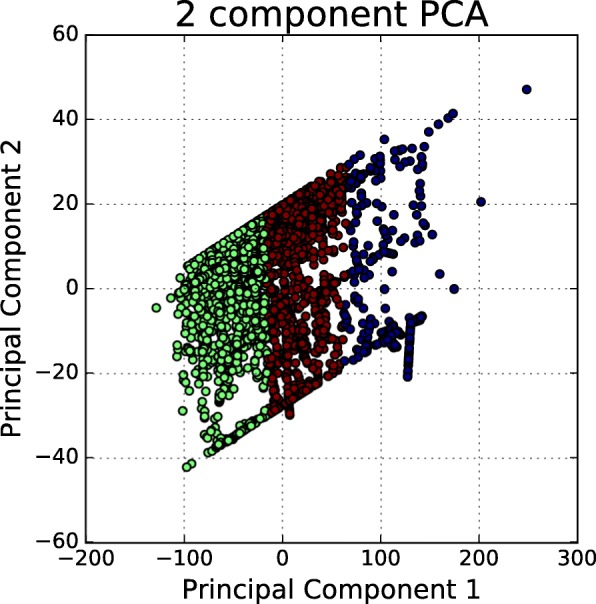
*K*-means Clustering

Figure 11 shows the elbow curve since after K=3, the elbow curve change slowly and remain less changing as compared to other K value which implies the addition of more clusters do not explain much more of the dataset. K=3 is the number of the reasonable cluster used in *k*-means clustering. The clustering result is showed in Fig. 12.

## Conclusion

In this study, we use the data-driven approach to analyzing the risk factors that contribute to MCI and weigh the importance of various factors. Using this approach, we re-affirm many previous discovered factors contributing to MCI such as depression, physical health, cigarette usage, education level, sleep time, kidney disease, alcohol consumption, and exercise. We also discover some other factors such as arthritis, pulmonary disease, stroke, asthma, and marital status that is less exploited previously. Using this novel approach, not only can we identify risk factors, but also we can weigh the importance of various factors. Among all the factors that we analyzed, we found depression disorder, physical health, mental health, arthritis, and stroke being the top five contributing factors to MCI. This data-driven approach can be expended to other medical record analysis and diagnosis area to accelerate the discovery of disease-disease correlation or disease risk factors.

## Data Availability

The public data used was collected from Centers for Disease Control and Prevention (CDC) based on Behavioral Risk Factor Surveillance System (BRFSS). The data download in https://www.cdc.gov/brfss/annual_data/annual_2016.html

## Abbreviations

AUC: Area under the ROC Curve
BRFSS: Behavioral risk factor surveillance system
CART: Classification and regression tree
CDC: Centers for disease control and prevention
MCI: Mild cognitive decline/ impairment (MCI)
ROC: Receiver operating characteristic
SMOTE: Synthetic minority over-sampling technique
SVM: Support vector machine
